# COVID-19 in Patients with Drug-Resistant Tuberculosis in Lesotho

**DOI:** 10.4269/ajtmh.23-0422

**Published:** 2023-10-09

**Authors:** Afom T. Andom, Meseret Asfaw, Llang Bridget Maama-Maime, Mikanda Kunda, Stephane Mpinda, Joia Mukherjee, Melino Ndayizigiye, Ninza Sheyo, Mosala Zulu, Carole D. Mitnick, Kwonjune J. Seung

**Affiliations:** Partners In Health, Maseru, Lesotho E-mail: aandom@pih.org; Partners In Health, Maseru, Lesotho E-mail: mtamirat@pih.org; Ministry of Health and Social Welfare, Maseru, Lesotho E-mail: maama36@hotmail.com; Partners In Health, Maseru, Lesotho E-mail: mkunda@pih.org; Partners In Health, Maseru, Lesotho E-mail: smpinda@pih.org; Harvard Medical School, Department of Global Health and Social Medicine, Boston, Massachusetts; Partners In Health, Boston, Massachusetts; and Brigham and Women’s Hospital, Boston, Massachusetts E-mail: jmukherjee@pih.org; Partners In Health, Maseru, Lesotho E-mail: mndayizigiye@pih.org; Partners In Health, Maseru, Lesotho E-mail: nsheyo@pih.org; Partners In Health, Maseru, Lesotho E-mail: mzulu@pih.org; Harvard Medical School, Department of Global Health and Social Medicine, Boston, Massachusetts; Partners In Health, Boston, Massachusetts E-mail: carole_mitnick@hms.harvard.edu; Harvard Medical School, Department of Global Health and Social Medicine, Boston, Massachusetts; Partners In Health, Boston, Massachusetts; and Brigham and Women’s Hospital, Boston, Massachusetts E-mail: kjseung@pih.org

Dear Sir:

We read with interest the short report “Coinfection of COVID-19 and Tuberculosis in Uganda,” which described the clinical presentation and outcomes of 11 tuberculosis (TB) patients diagnosed with COVID-19 during the Omicron surge.^1^ We agree with the authors that there are surprisingly few data about outcomes of COVID-19 in TB patients in sub-Saharan Africa. Here we present data from the national drug-resistant TB program in Lesotho, a country in southern Africa with an estimated incidence of TB of 650 per 100,000 (2020) and an estimated adult HIV positivity of 20.9% (2021).^2^

In Lesotho, there are approximately 200 rifampicin-resistant TB patients in treatment at any given time, and this number has remained largely stable for the past few years, even during the COVID-19 pandemic.^3^^,^^4^ Since the beginning of the COVID-19 pandemic, all patients are screened for symptoms of COVID-19 at all encounters at ambulatory clinics or hospitals and, if deemed appropriate, tested with a SARS-CoV-2 rapid antigen test (SD Biosensor, San Diego, CA).

From July 2020 to June 2022, 351 rapid antigen tests were done, of which 33 (9.4%) were positive (Table 1). Sixteen patients (48%) required hospitalization, oxygen, and corticosteroids. Four patients (12%) died during their hospital stay, similar to the hospitalized COVID-19 mortality rate reported in Uganda and other countries.^1^^,^^5^^,^^6^ Compared with the Ugandan cohort, a larger percentage of patients were HIV-positive (61%), as expected given the higher HIV seroprevalence in Lesotho. COVID-19 vaccines became available to the general population of Lesotho at the end of 2021, so most of the patients in this cohort were likely unvaccinated at the time of their SARS-CoV-2 infection.

**Table 1:** Characteristics and outcomes of 33 rifampicin resistant-tuberculosis patients diagnosed with COVID-19 in Lesotho

Male	21 (64%)
Age (median)	50.4 (37.1–63.1)
Comorbidities	
HIV infection	20 (61%)
Hypertension	9 (27%)
Asthma/chronic obstructive pulmonary disease	4 (12%)
Diabetes	1 (3%)
Symptoms of COVID-19*	
Cough	18 (55%)
Difficulty breathing	13 (39%)
Fatigue, weakness	9 (27%)
Chills/fever	8 (24%)
Upper respiratory symptoms (e.g., rhinorrhea, sore throat)	5 (15%)
None	2 (6%)
COVID-19 severity	
Received oxygen and corticosteroids	16 (48%)
COVID-19 outcome	
Died in the hospital from respiratory failure	4 (12%)
Died at home (cause of death unknown) after discharge	2 (6%)

* Most patients had typical symptoms of COVID-19, but six patients (18%) did not. One did not have any apparent infectious disease symptoms but was admitted for psychosis, which might have affected history taking. The other five patients had nonspecific symptoms: one patient was admitted for abdominal pain and drug-induced liver injury; one reported feeling worthless and tired (admitted for depression and suicidal ideation); one reported fatigue thought to be due to severe anemia; and two had runny/congested noses without other symptoms.

Two additional TB patients died several months after recovering from COVID-19. One patient, who had been asymptomatic from SARS-CoV-2 infection, died at home 2.5 months later of unknown causes. A second patient, who required oxygen, corticosteroids, and bronchodilators due to SARS-CoV-2 infection, was discharged after 2 months apparently fully recovered but then died at home 3 months later of unknown causes. Although the cause of death in these two patients is unknown, it is now recognized that even mild COVID-19 infection can increase the risk of thromboembolic events such as myocardial infarction or pulmonary embolism.^7^^,^^8^

In conclusion, in drug-resistant TB patients in Lesotho, COVID-19 illness was often severe or fatal, just as in drug-susceptible TB patients in Uganda. We agree with the authors that TB programs in low-income settings probably need to do a better job of screening for COVID-19. In our cohort, we found that COVID-19 often presented with symptoms indistinguishable from TB and common respiratory illnesses, so a high clinical suspicion and low threshold for SARS-CoV-2 testing is important. TB patients in all countries should be a high priority group for COVID-19 vaccination and boosting, as well as for access to antiviral drugs against SARS-CoV-2.
